# ImpACT+, a coping intervention to improve clinical outcomes for women living with HIV and sexual trauma in South Africa: study protocol for a randomized controlled trial

**DOI:** 10.1186/s13063-022-06655-5

**Published:** 2022-08-18

**Authors:** K. J. Sikkema, S. Rabie, A. King, M. H. Watt, M. I. Mulawa, L. S. Andersen, P. A. Wilson, A. Marais, E. Ndwandwa, S. Majokweni, C. Orrell, J. A. Joska

**Affiliations:** 1 Department of Sociomedical Sciences, Mailman School of Public Health, Columbia University, New York, NY USA; 2 Department of Psychiatry and Mental Health, HIV Mental Health Research Unit, Neuroscience Institute, University of Cape Town, Cape Town, South Africa; 3 Department of Population Health Sciences, University of Utah, Salt Lake City, UT USA; 4 Duke University School of Nursing and Duke Global Health Institute, Durham, NC USA; 5 Present Address, University of Copenhagen, Copenhagen, Denmark; 6 Department of Psychology, University of California, Los Angeles, USA; 7 Desmond Tutu HIV Foundation, University of Cape Town Medical School, Cape Town, South Africa

**Keywords:** HIV, Adherence, Antiretroviral therapy, Sexual violence, Traumatic stress, South Africa, Randomized controlled trial

## Abstract

**Background:**

Addressing sexual trauma in the context of HIV care is essential to improve clinical outcomes and mental health among women in South Africa. Women living with HIV (WLH) report disproportionately high levels of sexual trauma and have higher rates of posttraumatic stress disorder. Adherence to antiretroviral therapy (ART) may be difficult for traumatized women, as sexual trauma compounds the stress associated with managing HIV and is often comorbid with other mental health disorders, further compromising care engagement and adherence. ART initiation represents a unique window of opportunity for intervention to enhance motivation, increase care engagement, and address the negative effects of trauma on avoidant coping behaviors. Mental health interventions delivered by non-specialists in low- and middle-income countries have potential to treat depression, trauma, and effects of intimate partner violence among WLH. This study will examine the effectiveness of Improving AIDS Care after Trauma (ImpACT +), a task-shared, trauma-focused coping intervention, to promote viral suppression among WLH initiating ART in a South African clinic setting.

**Methods:**

This study will be conducted in Khayelitsha, a peri-urban settlement situated near Cape Town, South Africa. Using a hybrid type 1 effectiveness-implementation design, we will randomize 350 WLH initiating ART to the ImpACT + experimental condition or the control condition (three weekly sessions of adapted problem-solving therapy) to examine the effectiveness of ImpACT + on viral suppression, ART adherence, and the degree to which mental health outcomes mediate intervention effects. ImpACT + participants will receive six once-a-week coping intervention sessions and six monthly maintenance sessions over the follow-up period. We will conduct mental health and bio-behavioral assessments at baseline, 4, 8, and 12 months, with care engagement data extracted from medical records. We will explore scalability using the Consolidated Framework for Implementation Research (CFIR).

**Discussion:**

This trial is expected to yield important new information on psychologically informed intervention models that benefit the mental health and clinical outcomes of WLH with histories of sexual trauma. The proposed ImpACT + intervention, with its focus on building coping skills to address traumatic stress and engagement in HIV care and treatment, could have widespread impact on the health and wellbeing of individuals and communities in sub-Saharan Africa.

**Trial registration:**

Clinicaltrials.gov NCT04793217. Retrospectively registered on 11 March 2021.

## Administrative information

Note: the numbers in curly brackets in this protocol refer to SPIRIT checklist item numbers. The order of the items has been modified to group similar items (see http://www.equator-network.org/reporting-guidelines/spirit-2013-statement-defining-standard-protocol-items-for-clinical-trials/).

**Table:** 

Title {1}	ImpACT + , a coping intervention to improve clinical outcomes for women living with HIV and sexual trauma in South Africa: study protocol for a randomized controlled trial
Trial registration {2a and 2b}.	The trial was registered on the U.S. National Library of Medicine [ClinicalTrials.Gov] under the identifier NCT04793217 [registered after stary inclusion; March 11 2021]
Protocol version {3}	2 August 2022, Version 2
Funding {4}	National Institute of Mental Health (1R01MH118004-01A1)
Author details {5a}	Kathleen J. Sikkema, Department of Sociomedical Sciences, Mailman School of Public Health, Columbia University, New York, USA Stephan Rabie, HIV Mental Health Research Unit, Neuroscience Institute, Department of Psychiatry and Mental Health, University of Cape Town, Cape Town, South Africa Aisha King, Department of Sociomedical Sciences, Mailman School of Public Health, Columbia University, New York, USA Melissa H. Watt, Department of Population Health Sciences, University of Utah, Salt Lake City, UT, USA Marta I. Mulawa, Duke University School of Nursing, Durham, NC, USA; Duke Global Health Institute, Duke University, Durham, USA Lena S. Andersen, HIV Mental Health Research Unit, Neuroscience Institute, Department of Psychiatry and Mental Health, University of Cape Town, Cape Town, South Africa (Current address: University of Copenhagen, Copenhagen, Denmark) Patrick A. Wilson, Department of Psychology, University of California, Los Angeles, USA Adele Marais, HIV Mental Health Research Unit, Neuroscience Institute, Department of Psychiatry and Mental Health, University of Cape Town, Cape Town, South Africa Esona-sethu Ndwandwa, HIV Mental Health Research Unit, Neuroscience Institute, Department of Psychiatry and Mental Health, University of Cape Town, Cape Town, South Africa Sybil Majokweni, HIV Mental Health Research Unit, Neuroscience Institute, Department of Psychiatry and Mental Health, University of Cape Town, Cape Town, South Africa Catherine Orrell, Desmond Tutu HIV Foundation, University of Cape Town Medical School, Cape Town, South Africa John A. Joska, HIV Mental Health Research Unit, Neuroscience Institute, Department of Psychiatry and Mental Health, University of Cape Town, Cape Town, South Africa
Name and contact information for the trial sponsor {5b}	Kathleen Sikkema, Department of Sociomedical Sciences, Mailman School of Public Health, Columbia University, New York, USA Email: ks3364@cumc.columbia.edu
Role of sponsor {5c}	This is an investigator initiated trial. The study funder played no role in the study design, data collection, analysis, or its interpretation, nor in writing this manuscript.

## Introduction

### Background and rationale {6a}

Addressing sexual trauma in the context of HIV care is essential to improve clinical and mental health outcomes. Women living with HIV (WLH) report disproportionately high levels of sexual trauma and have high rates of posttraumatic stress disorder (PTSD) [1, 2]. Adherence may be difficult for traumatized women, as sexual trauma adds to the stress of managing HIV and increases avoidant behavior [3–7], acting as a barrier to care engagement [4, 8] and resulting in compromised immune functioning and increased infectivity [9–16]. Addressing the synergy of mental health and behavioral factors may be an effective strategy to improve adherence and clinical outcomes from the outset of ART initiation [9, 15, 16]. With increased global access to highly effective antiretroviral treatment (ART) that has resulted in viral suppression as an achievable goal, innovative intervention strategies are needed to address underlying causes of treatment failure and build resilience to maintain adherence [17].

In South Africa, the country with the highest burden of HIV, nearly half of all women experience physical or sexual assault from a male partner in their lifetime [5, 18, 19] and over a third have histories of sexual abuse during childhood [20]. These instances of violence frequently result in high levels of post-traumatic stress among WLH [21–23]. The experience of sexual trauma and HIV may be linked either directly (acquiring HIV through sexual abuse or violence) or indirectly (through behavioral sequelae, disclosing to a violent partner, or internalized stigma) [24]. Traumatic stress is often comorbid with other mental health disorders, such as depression, anxiety disorders, and substance use [25–27], which are also linked to poor care engagement [28, 29]. The high burden of sexual trauma among WLH in South Africa [19, 30, 31] may therefore be a significant contributing factor for the low rates of viral suppression seen among WLH [32].

The intersection of HIV and trauma highlights the importance of integrated mental health treatment and HIV care, particularly in a setting like South Africa where mental health services are limited, and women are both more likely to be living with HIV [33] and face overlapping epidemics of gender-based violence and sexual trauma [34]. Specifically, effective interventions are needed that address the combined stress of trauma and HIV among WLH to improve psychological and clinical outcomes from the outset of ART initiation [35, 36]. There is strong reason to expect that reducing traumatic stress would decrease avoidance [37, 38] and increase care engagement [1], thus increasing viral suppression. Although some interventions aimed at treating sexual trauma among WLH in order to improve care engagement have been developed [1, 37–39], and treatments aimed at addressing IPV in this population have shown promise [40], we are unaware of any full-scale trials that test the effectiveness and examine the underlying mechanisms of change of a trauma-related coping intervention to improve or maintain viral suppression and related clinical outcomes.

ART initiation represents a unique window of opportunity to enhance motivation and address the negative effects of trauma on avoidant coping behaviors [41, 42] to increase care engagement and interrupt losses that occur throughout HIV care cascade [43, 44]. In 2016, we conducted a pilot trial of ImpACT, a task-shared intervention that sought to improve mental health and clinical outcomes among WLH with sexual trauma histories in South Africa by addressing coping with HIV and trauma and therefore increasing adherence to anti-retroviral therapy (ART) [45, 46]. The ImpACT intervention was based on *Living in the Face of Trauma* (LIFT), a CDC—and SAMHSA—evidence-based intervention for adults living with HIV with histories of childhood sexual abuse in the United States (US). LIFT significantly reduced incidents of unprotected sex [47], substance use [48], and traumatic stress symptoms [37]. Notably, reductions in traumatic stress were fully explained by reductions in avoidant coping. ImpACT was adapted and refined to become ImpACT + and is the intervention evaluated in this trial. Task-shared for delivery by a non-specialist, ImpACT + shows promise to address the synergistic stress of sexual trauma and HIV and the multifaceted needs of WLH in South Africa. In this trial, we aim to prove the efficacy of this acceptable, feasible, and culturally relevant intervention to improve clinical outcomes.

The paucity of culturally relevant evidence-based mental health treatments is accompanied by two significant barriers to scalability: (1) the shortage of trained mental healthcare providers across low resource settings [49] and (2) uncertainty as to the best setting for delivery [50]. There is increasing evidence that culturally adapted mental health interventions delivered by nonspecialists in LMICs have potential to treat depression, trauma, and effects of intimate partner violence (IPV) among WLH [40, 45, 51]. Care providers in LMICs emphasize the need for mental health interventions that can be integrated into chronic care systems and strengthen the healthcare system [52]. Integrating a targeted, task-shared trauma intervention into HIV care may help address the limited capacity of the mental health care system to identify and treat women with traumatic stress.

The current study will examine the effect of ImpACT + , a task-shared, trauma-focused intervention at promoting viral suppression among WLH initiating ARVs in a South African clinic setting. We will also examine whether the intervention’s effects are mediated by improvements in mental health and coping outcomes. The study design will allow us to explore the potential for implementation and scale-up of ImpACT + , thereby accelerating the process of translating research into practice.

### Objectives {7}

The primary objective of this trial is to determine the effectiveness of ImpACT + on viral load suppression by reducing avoidant coping and traumatic stress among WLH with sexual trauma in Cape Town, South Africa.

The specific objectives are:

1. To determine the effectiveness of ImpACT + , compared to adapted problem-solving therapy (PST), on clinical outcomes over a 12-month period among WLH with sexual trauma who are initiating ART
2. To examine underlying mechanisms of change by evaluating the degree to which improvements in mental health and coping mediate intervention effects on primary (viral suppression) and secondary outcomes (adherence and care engagement)
3. To explore the scalability of ImpACT + , guided by the Consolidated Framework for Implementation Research (CFIR) [53], to better understand facilitators and barriers to full-scale implementation

Our primary hypothesis is that a greater proportion of ImpACT + participants will be virally suppressed (primary outcome), adherent, and engaged in care (secondary outcomes) at 12 months compared to PST participants. Our secondary hypothesis is that over time, ImpACT + participants, as compared to PST participants, will report greater reductions in (1) traumatic stress and (2) avoidant coping, which, in turn, will lead to (causally mediated) improvements in ART adherence, care engagement, and viral load (VL) at 12 months.

### Trial design {8}

**Fig. 1:**
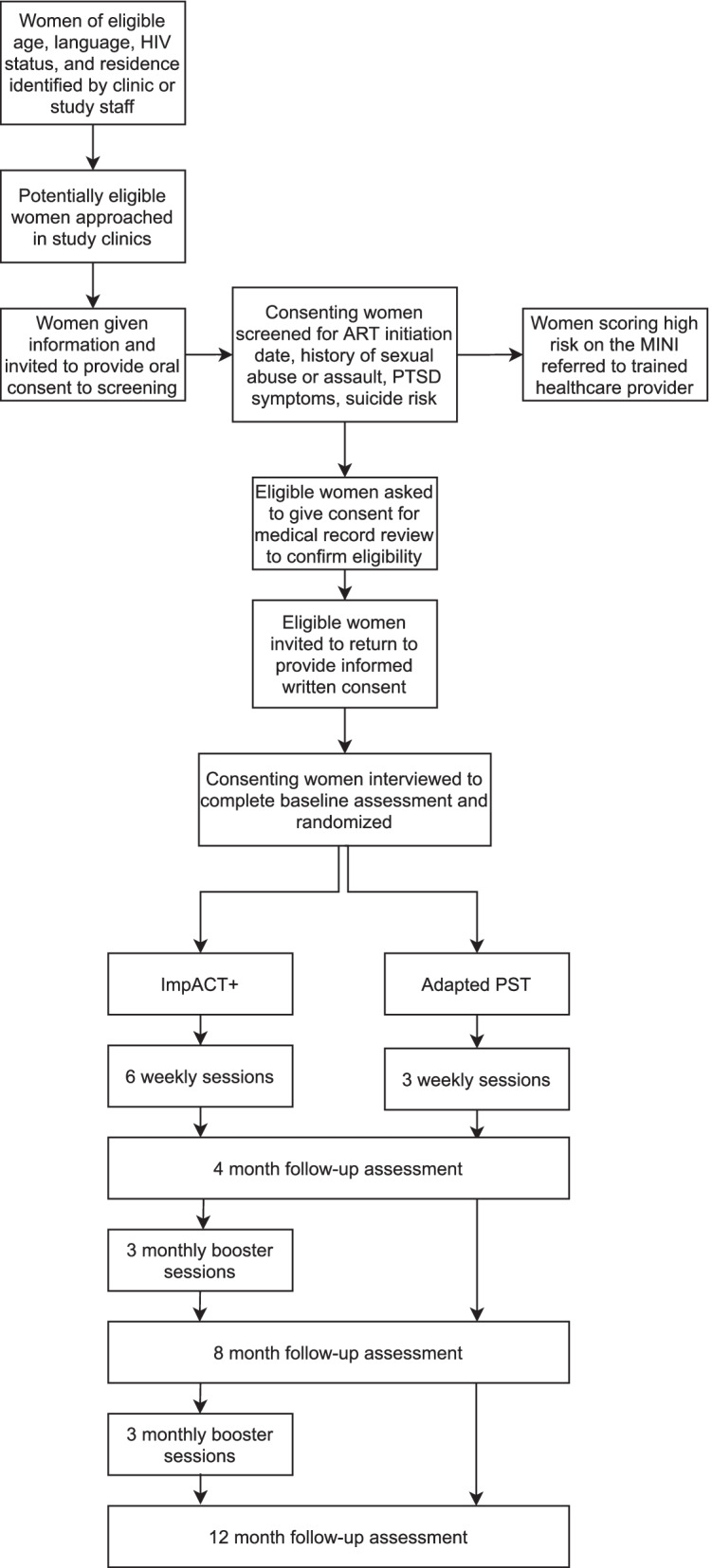
Study flow

We will employ a hybrid type 1 effectiveness-implementation design [54] to test the effectiveness of ImpACT + and explore its potential for implementation. This trial is a two-arm, randomized controlled trial comparing ImpACT + to adapted PST among WLH with sexual trauma (see Fig. 1 for study flow). Both arms enhance standard clinical care.

## Methods: participants, interventions, and outcomes

### Study setting {9}

The study will be conducted in four primary healthcare facilities located in the peri-urban suburb of Khayelitsha, situated on the outskirts of Cape Town, South Africa. Khayelitsha is one of the fastest growing informal areas of South Africa, with a conservatively estimated population of 500,000 [55]. Khayelitsha is the region with the highest HIV prevalence in the Western Cape [56] and is characterized by high rates of unemployment and poverty, with almost half of residents living in overcrowded, poor quality housing without electricity, indoor sewage, and/or running water [57]. The healthcare facilities offer comprehensive HIV care, and staff are comprised of nurses, adherence counselors, community care workers, pharmacists, medical officers, and social workers. Each of the four facilities serves between 7500 and 10,000 patients living with HIV, of which two-thirds are female and a majority are Black African and isiXhosa-speaking. Universal test and treat guidelines were introduced in South Africa in 2016, and ART initiation at the clinic follows the standard protocol per government guidelines [58]. The ART initiation clinics serve naive initiators (often newly diagnosed), restarters (absent from care ≤ 3 months), and defaulters (absent from care > 3 months).

### Eligibility criteria {10}

The study will enroll women living with HIV engaging with care at one of the four study clinics. Women will be eligible for enrollment in the trial if they meet the following criteria: (1) Living with HIV and between 2 weeks and 4 months since initiation of first-line ART (as new initiators, restarters or defaulters); (2) history of sexual abuse or assault during childhood, adolescence, or adulthood, using four items based on the WHO CIDI [59] and the Childhood Trauma Questionnaire [60]; (3) endorsement of any PTSD symptoms as assessed by the Breslau PTSD screener [61]; (4) 18 years or older; (5) isiXhosa speaking; and (6) receiving HIV care services at a study clinic. Patients will be excluded and referred to psychiatric services if they meet criteria for high risk of suicide. Suicidal ideation and severity will be assessed using items adapted from the suicidality subscale of the Mini International Neuropsychiatric Interview [62]. Pregnant women will be eligible to enroll. Additional exclusion criteria include patients initiated on second- or third-line ART, the inability to provide informed consent, or inability to communicate in isiXhosa or English.

### Who will take informed consent? {26a}

Prior to baseline assessment, study staff will provide participants with verbal and written information about the study in their preferred language (English or isiXhosa). Participants will be asked to provide written consent if they agree to be part of the study. Participants will sign two copies of the informed consent form, one for the study records and one to take for their personal documentation.

### Additional consent provisions for collection and use of participant data and biological specimens {26b}

We will request consent for review of participants’ medical records, and for the collection of blood samples to assess viral load and adherence.

### Interventions

#### Explanation for the choice of comparators {6b}

ImpACT + was developed for a South African clinic setting in the context of limited mental health resources and with national guidelines indicating rapid ART initiation and universal treatment for all HIV-infected persons. Integrating a targeted trauma intervention into HIV care may help address the limited capacity of the mental health care system to identify and treat women with traumatic stress. For the control condition, we decided to provide another mental health treatment (adapted PST) rather than standard of care for mental health treatment (referral to the clinic medical officer). This decision was based primarily on the following: (1) ethical grounds, to provide a reasonable comparison intervention for this vulnerable patient population; PST is being explored in an implementation trial in Cape Town [63, 64], and (2) to utilize a control condition that provides a relatively high level of care and accounts for the potential impact of general stress reduction, thus, setting a high standard for determining the effectiveness of ImpACT + . With the absence of trauma-informed care in this setting, our research process identifies women with sexual trauma that may otherwise go unaddressed. A control condition with minimal interaction, or even treatment as usual, is insufficient. We have chosen this control condition because we believe that the greatest priority is to measure the effectiveness of ImpACT + in comparison to recommended mental health care services, even if they have not yet been brought to scale in the settings.

#### Intervention description {11a}

## Intervention arm

*Treatment condition: ImpACT* + *.* ImpACT + integrates skills for coping with trauma and HIV treatment adherence, tailored to the South African context. Culturally adapted and guided by the evidence-based intervention *Living in the Face of Trauma* (LIFT) [37, 47, 48], ImpACT + targets women who are initiating ART, making use of a window of opportunity to maximize the impact on HIV care engagement. ImpACT + draws on LIFT as a conceptual foundation [65] and the pilot trial of ImpACT for cultural adaptation [46, 66]. Areas of focus include exploration of values informing care engagement, recognizing the synergistic stress of sexual trauma and HIV, understanding the contribution of stressors to maladaptive coping, and developing adaptive methods for coping (including disclosure) as alternatives to avoidance. Key intervention components were developed for cultural saliency. For example, a “coping pebbles” activity was used to translate the coping framework [67, 68] into a tangible analogy to represent the burden of a global stressor, with stress appraisal using color-coded pebbles to distinguish changeable and unchangeable stressors. Concepts from LIFT related to safety, trust, power, and self-esteem were symbolized by the locally relevant *Imbiza pot*, with three legs and a lid representing these concepts. Intervention materials developed include a detailed manual, workbook, and flipbook for in-session use. See Table 1 for key conceptual concepts.

**Table 1:**
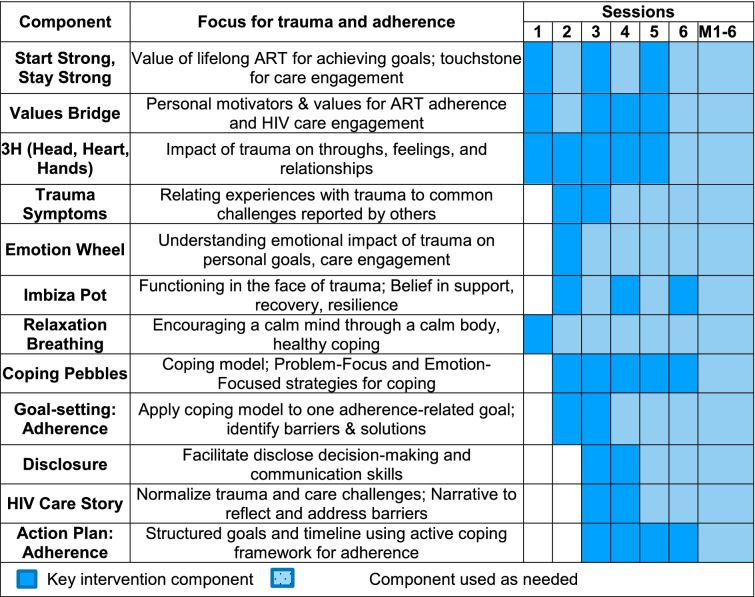
ImpACT + components and timing

ImpACT + will be delivered in private, study offices at each research site and will consist of six weekly (over 2 months) structured individual sessions lasting 60 min, followed by six monthly maintenance check-ins lasting 30 min linked to routine appointments. Sessions will focus on motivational enhancement, coping skills related to HIV and trauma, adherence, and care engagement during a critical period of initiation, while maintenance check-ins will reinforce positive change and support the ongoing implementation of skills as new challenges arise. Evidence supports a six-session format, with interventions of similar length shown to be effective in low-resource settings [64, 69], and maintenance check-ins for longer term effects [68, 70, 71]. Individual sessions will begin within 2 weeks after the baseline survey, and maintenance check-ins will begin following the 4-month assessment.

*Personnel: recruitment, training, and supervision.* The ImpACT + intervention will be conducted by trained and supervised healthcare providers with a 4-year degree or diploma program, including relevant mental health training or prior experience in providing care or support to women with HIV and/or trauma histories. Although this is a task-shared intervention, the trauma-focused nature of the intervention indicates that some prior experience in mental health care is necessary to ensure the mental wellbeing of the interventionists. Interventionist training will be conducted over 5 weeks; the first 2 weeks of intensive face-to-face training will focus on learning the ImpACT + manual and workbook, followed by 3 weeks of practice administering individual components, mock sessions, and training on ethical conduct of counseling. Training will be ongoing throughout the trial, particularly in the first 3 months of intervention delivery. Additionally, interventionists will engage in weekly group supervision throughout the trial which will include review and feedback on randomly selected session recordings (10%, stratified by interventionist and session number), review of session workbook notes, and quality assurance checklists. Supervisors will use an adapted version of the Enhancing Assessment of Common Therapeutic factors (ENACT) rating scale to rate sessions and determine fidelity [72]. Interventionists will furthermore participate in monthly debriefing sessions with a trained clinical psychologist who specializes in treating traumatized women. Additional debriefing sessions will be available upon request.

## Control arm

*Control condition*: *adapted PST*. Participants randomly assigned to the control condition will receive three, 40-min individual sessions of adapted PST. The version of PST developed for the purposes of this study is based on Problem Management Plus (PM +), a component of the open-access WHO Mental Health Gap Action Programme (mhGAP) [73]. PST is a psychoeducational treatment focused on managing the negative effects of stressful life events. PST has been found to be effective for a range of problems, such as depression, and is recommended for implementation in low-resource settings. The goal of PST in this study is to identify problems that interfere with daily activities and address them through problem-orientation work. We anticipate stressors will include (a) relationship difficulties, including family stress, (b) financial stress and unemployment, (c) general impact of HIV infection, and (d) overall chronic stress. Thus, PST is stress management focused, but will not address the intersection of HIV and trauma specifically. PST interventionists will not prevent participants from discussing sexual trauma and HIV, but the stress and coping skills training related to the synergy between trauma and ART adherence will not be incorporated into the PST interventionist training. These procedures will maintain the distinction between the ImpACT + intervention and the control condition. Participants randomized to the PST condition will begin sessions within 2 weeks post-baseline.

*Personnel: recruitment, training, and supervision.* Adapted PST will be conducted by a trained and supervised lay counsellor. The PST counsellor will have at minimum a secondary school education and experience in providing counselling to women living with HIV. Intervention training will be conducted over a 4-week period, with two weeks of intensive face-to-face training using an adapted PST manual and workbook developed by study research staff, followed by 2 weeks of practice administering intervention sessions, conducting mock sessions, and learning basic counselling skills and therapeutic conduct. Supervision is similar to that of the ImpACT + interventionists. The PST counsellor engages in weekly one-on-one supervision for review of a random subset of sessions, session workbooks, and ENACT forms. The PST counsellor participates in monthly debriefing sessions separate from the ImpACT + interventionists to mitigate risk of intervention contamination.

### Criteria for discontinuing or modifying allocated interventions {11b}

Intervention sessions will be discontinued if participants request to withdraw from the study. Participants may be withdrawn by the investigator in the event of a serious adverse event that precludes participation in the intervention.

### Strategies to improve adherence to interventions {11c}

The ImpACT + intervention manual was designed to allow the interventionists to tailor content to traumatic experience, stress symptoms, and adherence challenges depending on the personal history and mental health state of participants. As such, the intervention will vary between participants and adherence to the manual will be high. In the event that participants are unable to attend one or several sessions of the intervention, the following sessions will be adapted to include key components missed. Following each session, ImpACT + interventionists and PST counsellors will complete a quality session-specific fidelity checklists and note any issues that arose. ImpACT + and PST providers will engage in separate weekly group supervision, including review and feedback on select audio recordings of sessions, and review of session workbook notes and quality assurance checklists. Each week, supervisors will listen to a random selection of session audio recordings, using the ENhancing Assessment of Common Therapeutic factors (ENACT) rating scale to assess therapeutic competence and intervention fidelity [72].

### Relevant concomitant care permitted or prohibited during the trial {11d}

Mental health services outside of protocol will be monitored throughout the trial.

### Provisions for post-trial care {30}

There are no plans for post-trial care. Potential participants will be informed of the risks associated with participating in the trial. Referrals will be made if deemed necessary and appropriate.

### Outcomes {12}

The primary outcome measure is viral suppression at 12 months, determined by HIV viral copies per milliliter of blood. Viral suppression will be reported as a dichotomous variable with the threshold for viral suppression defined as < 50 copies/mL, as determined by the Abbott Alinity assay (Sensitivity Limit of Detection 20 copies/mL).

The following secondary outcome measures will be assessed at baseline, 4, 8, and 12-month timepoints:

- ART adherence:
  - ◦ Dried blood spots measuring levels of tenofovir-diphosphate (TFV-DP) (at 12 months)
  - ◦ Medical record abstraction of pharmacy visits and pharmacy refill data
  - ◦ Adapted Adult AIDS Clinical Trials Group self-report measure of adherence [74, 75]
- HIV care engagement (medical record abstraction):
  - ◦ Missed visits (# of monthly visits missed and binary indicator of any missed monthly visit)
  - ◦ Visit adherence (proportion of visits kept)
  - ◦ Gaps in care (whether > 90 days have elapsed between visits)
  - ◦ Visit constancy (# of 90-day intervals with > 1 completed visit).
    ◦ Mental health mediators will be assessed by the following measures (all timepoints):
- Post-Traumatic Stress (PTSD Checklist-Civilian version for the DSM-5 (PCL-5) [76]
- Coping [45], adapted from [77–81].

### Implementation and scalability

In addition to examining the efficacy of ImpACT + , this trial will include a mixed-method process evaluation of intervention feasibility and acceptability. We will explore the scalability of ImpACT + by assessing potential facilitators and barriers to full-scale implementation, guided by the CFIR [53]. The CFIR helps evaluate an intervention’s effectiveness in a specific context and identify strategies to optimize intervention benefits. We will draw on these CFIR domains: (1) intervention characteristics, (2) outer setting, (3) inner setting, and (4) process. After recruitment is complete, we will conduct in-depth qualitative interviews with clinic staff (*n* = 10 per clinic, 20 total: nurses, physicians, social workers, adherence counsellors, community health workers) and ImpACT + interventionists to explore intervention factors that would facilitate or impede roll-out and the sustainability of implementation. Interviews with clinic staff and managers at the City of Cape Town Health Department (*n* = 10) will explore patient needs and resources, and external policies and incentives that could impact future implementation and scalability. A random subset of ImpACT + participants (10%; *n* = 18) will be asked to participate in an in-depth interview on acceptability of the intervention and barriers and facilitators to implementation.

**Table 2:**
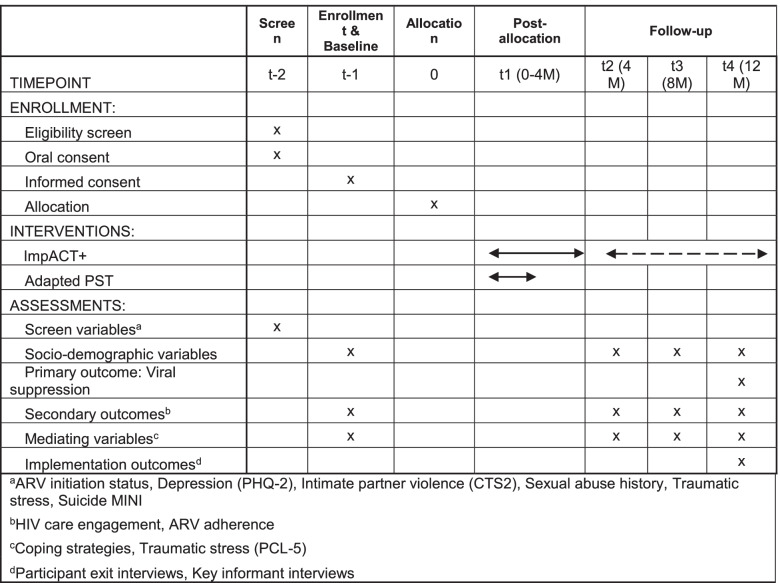
Schedule of enrollment, interventions, and assessments

### Participant timeline (Table 2) {13}

#### Sample size {14}

Our enrollment target is 350, which will allow detection of intervention effects on viral suppression at 12-month follow-up. Based on data from our preliminary studies [66] and corroborated by national estimates [32], we expect that 57% of women who are newly initiating ART and randomized to the PST condition will be virally suppressed at the 12-month follow-up. With 350 women randomized equally to both conditions, we will have 80% power with a two-tailed alpha level of 0.05 to detect differences between a proportion of 57% in the PST condition and 73% in the ImpACT + condition. This power calculation assumes we will retain 80% of participants over the study period. In the pilot, we were able to successfully retain 80% of all participants (83.3% of newly initiating participants) for the duration of the study [45].

#### Recruitment {15}

Clinic staff will refer all women who receive a positive HIV diagnosis or present themselves for care at the ARV initiation clinic to our study offices. In a private office, English and isiXhosa-speaking study staff will greet potential participants and ask if they would like to participate in a screening questionnaire. Prior to screening, study staff will administer oral consent that highlights confidentiality and enumerates the sensitive nature of the screening items. As receipt of an HIV diagnosis and disclosure of traumatic sexual experiences can both be distressing, study staff will judge whether newly diagnosed women should be screened same day or at her next clinic visit. Our window period of 2 weeks to 4 months post ART initiation allows for flexibility in recruitment based on individual participant needs.

Screening consists of three major sections: (1) oral consent for screening; (2) a questionnaire that includes six questions on demographics, three questions on interpersonal violence (IPV), four questions on sexual trauma, two questions on depression, and seven questions on traumatic stress symptoms; and (3) written consent for medical record access in order to confirm eligibility. Eligible participants will be given information about the trial and, if interested, scheduled for a baseline assessment. Participants who indicate high risk of suicide during screening will be assessed by on-site study interventionists to determine need for referral for additional mental health care. The project manager and clinical supervisor will confirm the interventionist’s assessment and determine whether the participant should be excluded.

## Assignment of interventions: allocation

### Sequence generation {16a}

1:1 allocation to either the intervention or control condition will be determined by a random number chart generated using an independent web-based online system (https://www.sealedenvelope.com). Randomization will be stratified by initiation status (naïve initiator, restarter, defaulter), clinic, and pregnancy status (pregnant, not pregnant) to ensure equal distribution of study conditions.

### Concealment mechanism {16b}

Randomized blocks of 4 and 6 will be used within the study sites to ensure that assessment staff are unaware of condition prior to allocation and to ensure balanced assignment to condition over time.

### Implementation {16c}

Study staff at Columbia University and Irving Medical Center will create the allocation table and upload it to REDCap. Following completion of the baseline assessment, study staff will use the Research Electronic Data Capture (REDCap) [82] randomization form to assign the participant to either ImpACT + or the PST condition. Participants will be informed that they have been assigned to either a 6-session or 3-session intervention and that they will receive further details at the first treatment session.

## Assignment of interventions: blinding

### Who will be blinded {17a}

Given differences in content and number of sessions, the experimental (ImpACT +) and control (PST) providers delivering the intervention cannot be masked to participant allocation. To avoid contamination, ImpACT + and PST providers will be trained separately and receive supervision by two distinct psychologists with separate debriefing sessions throughout the trial. Research assistants will inform participants of their intervention allocation post-baseline and are therefore not blinded post randomization. An independent assessor blinded to participant condition will conduct 4-, 8-, and 12-month assessments. The independent assessor will be trained, supervised, and attend debriefing sessions separately throughout the trial. Investigators, data analysts, and outcome assessors will be masked to participant allocation, with condition revealed at the conclusion of analysis.

### Procedure for unblinding if needed {17b}

Randomization allocation will be revealed to the statistician after finalization of the main data analysis.

## Data collection and management

### Plans for assessment and collection of outcomes {18a}

Participants will go through the full trial informed consent process at baseline, after which RAs will verbally administer a battery of psychological assessments and other self-report measures in isiXhosa. Independent assessors will administer the same battery at 4-, 8-, and 12-month follow-up assessments. Assessments will take approximately 60 min and will be conducted in a private room at the clinic. Study staff will clarify any questions and refer participants who indicate high suicide risk during the interviews to the medical officers for further assessment and referral. Blood samples to measure viral load and ARV adherence via DBS will also be collected at all follow-up assessments. Relevant HIV care data will be abstracted from participants’ medical records.

### Plans to promote participant retention and complete follow-up {18b}

Study staff will emphasize the importance of attending all intervention sessions and assessment appointments. We will collect detailed contact information for participants and close contacts. We will reschedule appointments as needed and follow-up with participants who miss sessions and assessments.

### Data management {19}

The PI and UCT PI, CUIMC Project Coordinator, and UCT Project Director will oversee the data management protocol and established Standard Operating Procedures (SOPs) for data collection, quality control, and data extraction and transfer. See the confidentiality section for precautions and quality assurance measures designed to protect the privacy of participants and maintain confidentiality of research data during collection and transfer. To ensure compliance with the monitoring plan, all study staff will be well trained and will receive ongoing supervision in confidentiality and data security procedures, specifically in ethical conduct, confidentiality protection, review of medical records, mandated reporting, data reporting across study sites, and other topics of human participant protection.

Surveys will be administered via password-protected, encrypted tablet computers by individual interview and entered in real time into REDCap projects. Screening, baseline, and follow-up assessments will be conducted in person. Survey data collected on the tablet will be coded with the participants’ ID numbers and will not contain participant names or contact information.

### Confidentiality {27}

Study staff will be trained and will receive ongoing supervision in confidentiality and data security procedures. As part of the consent procedure, participants will be informed of the limits of confidentiality (i.e., reporting of imminent harm to self or others) and mandated reporting requirements (i.e., reporting of child abuse). Interviews, discussions, study assessments, and intervention sessions will be conducted in closed and private rooms on password-protected tablets. Participant information will be stored on password-protected computers or locked cabinets and will not be linked to participant names or locator data. Audio files from intervention sessions will be deidentified and audio-translated into English for quality assurance purposes, and the original files will be deleted upon completion of all analyses. Data will be stored for as long as necessary to complete the study and for adherence to IRB regulations. Biospecimens will be de-identified and destroyed 5 years after study completion. The information gathered in this study will be used only for scientific, educational, or instructional purposes.

### Plans for collection, laboratory evaluation, and storage of biological specimens for genetic or molecular analysis in this trial/future use {33}

Trained personnel will take the blood samples, using only new, sterile equipment, and bandage the site to minimize any potential side effects. Staff will have received prior training in phlebotomy and will review best practices recommended by WHO guidelines on drawing blood. Blood samples will be assigned a unique study ID number and will be stored securely in a locked cabinet at the clinic until they can be transported in a batch to the laboratory for testing. Results will be confidential, following the same procedures as other study data. Viral load results are identifiable only by a study number. Laboratory staff will make results available on a password-enabled, HIPAA-compliant website and will communicate by direct e-mail to the study coordinator.

## Statistical methods

### Statistical methods for primary and secondary outcomes {20a}

To assess intervention effects on the primary outcome (viral suppression), we will compare the proportion of participants achieving HIV viral suppression at 12 month follow-up across study conditions by estimating a risk ratio and obtaining confidence intervals using robust error variances with a modified Poisson model and log link [83]. We will similarly obtain risk ratio estimates to assess intervention effects on the secondary outcomes (i.e., measures of ART adherence and care engagement) using the modified Poisson approach appropriate distribution and link function based on the outcome examined.

To examine the underlying mechanisms of the intervention effects on primary and secondary outcomes at 12 months, we will evaluate the effects of ImpACT + , compared to PST, on hypothesized mediators (e.g., levels of traumatic stress and avoidant coping). Our analytic approach will incorporate the appropriate distribution and link function depending on the distribution of these mediators. We will then examine the effect of both mediators on viral load. If the product of these two paths is greater than zero, this will serve as evidence of mediation [84]. The same approach will be utilized to evaluate the degree to which changes in traumatic stress and avoidant coping mediate intervention effects on secondary outcomes (ART adherence and care engagement).

Finally, qualitative data will be analyzed using a thematic analysis approach [85]. Memos will be written for each transcript to synthesize the emerging themes across the CFIR domains. Themes will be identified via consensus discussion after independent review of transcripts and textually coded using NVivo and/or ATLAS.ti software by multiple coders. Data display matrices will be used to examine commonalities and differences across informants [86]. A diagrammatic visual, overlaid on the CFIR framework [53] will highlight the potential barriers and facilitators to full-scale implementation.

### Interim analyses {21b}

No additional analyses are planned.

### Methods for additional analyses (e.g., subgroup analyses) {20b}

No additional analyses are planned.

### Methods in analysis to handle protocol non-adherence and any statistical methods to handle missing data {20c}

All analyses will be based on the intention-to-treat (ITT) principle such that participants will be analyzed in the arm to which they were randomized, regardless of intervention exposure. We will perform sensitivity analyses to address loss to follow-up (LTFU). We will summarize baseline characteristics by LTFU status separately by study conditions to identify possible predictors of LTFU and whether they differ by condition. Covariates that are predictive of LTFU will be included in regression models to assess any changes to conclusions based on an assumption of missing-at-random dependent on covariates. Pattern mixture approaches will be used to explore possible missing-not-at-random mechanisms [87].

### Plans to give access to the full protocol, participant-level data, and statistical code {31c}

The primary investigators, project coordinator at CUIMC, project director at UCT, and statisticians at CUIMC will be given full access to trial data.

## Oversight and monitoring

### Composition of the coordinating center and trial steering committee {5d}

A formal coordinating center is not needed for this single-site study.

Daily support for the trial is provided by:

Principal Investigators: provides scientific oversite and responsibility.

Clinical supervisor: supervises nonspecialists interventionists.

Clinical psychologist: emotionally supports field staff.

Project director: oversees daily trial activity and supervises control.

Senior research assistant: supervises research assistants and field data collection.

Project coordinator: organizes data collection, ensures data quality, trial registration, and annual reports.

Research assistants: take informed consent, administer assessments, and schedule appointments.

The study team meets weekly.

### Composition of the data monitoring committee, its role, and reporting structure {21a}

Throughout the course of the trial, all protocol modifications will be reported to the University of Cape Town’s Human Resources and Ethics Committee (HREC), Columbia University IRB, and the Data Safety and Monitoring Board (DSMB). The DSMB is comprised of independent, experienced, and highly qualified members who are free of any professional or financial conflict of interest with the study project and investigators. The DSMB reviewed the study protocol and data monitoring procedures prior to trial recruitment and approved the data monitoring plan for the remainder of the study. The DSMB will meet on an annual basis to review study progress, enrollment, randomization, and retention data (including reasons for dropout and retention by study condition); data integrity; and patient safety data, including reported adverse events and protocol deviations.

### Adverse event reporting and harms {22}

All adverse events will be recorded. We will follow the reporting guidelines as set out by UCT HREC, CUIMC IRB, NIMH, and our DSMB.

### Frequency and plans for auditing trial conduct {23}

CUIMC IRB and UCT HREC will meet annually to review trial progress and conduct throughout the trial period. In addition, all adverse events and protocol deviations will be reported according to IRB And HREC guidelines.

### Plans for communicating important protocol amendments to relevant parties (e.g., trial participants, ethical committees) {25}

We will seek approval from CUIMC and UCT HREC prior to implementing any protocol amendments, and to NIMH when required.

### Dissemination plans {31a}

In addition to academic manuscripts and presentations, we will collaborate with the City of Cape Town and HIV and policy makers to disseminate lessons learned in our research. If effective, we will work towards full-scale implementation of ImpACT + in the region and be available to adapt the intervention for other global settings.

## Discussion

There is a substantial treatment gap for women living with HIV (WLH) who have experienced sexual trauma. The scientific evidence on the coexisting burdens of HIV infection and sexual trauma among women in South Africa, and the well-documented drop-off from linkage to HIV care to viral suppression across the HIV care cascade [32, 43, 88] demonstrate the need for evidence-based, scalable interventions that address HIV care engagement in this context. Specifically, there is an urgent need to address the psychological sequelae of HIV, sexual trauma, and mental health, which can significantly impact HIV treatment adherence.

This trial is unique for several reasons. Firstly, despite robust evidence highlighting the adverse impact of sexual trauma on HIV treatment adherence and care engagement, we have not identified any full-scale randomized controlled trials demonstrating the effectiveness of trauma-focused psychological and behavioral interventions to improve biological outcomes in South Africa, or other low- and middle-income countries (LMIC) [89, 90]. This study aims to determine whether a mental health intervention to reduce avoidant coping and traumatic stress in comparison to a high-quality control intervention condition can improve clinical HIV outcomes, including viral suppression (primary outcome) and ART adherence and HIV care engagement (secondary outcomes). This type of holistic approach is not only key to improve individual outcomes, but also to motivating health departments to increase resources dedicated to integrating mental health services into chronic disease settings. As the revised UNAIDS care cascade goals move towards even higher rates of ART initiation, care retention, and viral load suppression, health systems will need to develop ways to address important behavioral and psychosocial determinants of treatment failure.

The second important innovation is that this study will explore whether increased likelihood of viral suppression is mediated by improvements in mental health. Demonstrating that improvement in psychological symptoms mediates the effectiveness of a behavioral intervention to improve adherence will strengthen the case for mental health services to be integrated in primary health care settings, particularly in the context of large mental health treatment gaps in LMICs [91]. In the absence of adequate numbers of trained psychologists and other mental health specialists, the use of culturally appropriate, manualized, and task-shared approaches becomes even more compelling. Given the sensitivity required to broach topics of sexual trauma and the potential for vicarious trauma, ImpACT + is intended for delivery by trained and supervised interventionists. Nurses and social workers are frequently employed in primary healthcare settings and are therefore the most likely candidates for interventionists. These cadres represent a key resource in the South African primary health care system, as well as in many other LMIC.

The third innovation is that we will report on critical implementation outcomes within the context of primary health care. Qualitative data from interventionists, facility managers, local stakeholders, and WLH with lived experience of trauma will inform potential and plans for implementation. Therefore, this trial will systematically evaluate feasibility and acceptability in addition to primary effectiveness. The implementation outcomes of this study will be presented to key stakeholders for consideration for adoption in health system training and capacitation programs. Our goal is to contribute to future policy and practice in the wider health care system.

Despite the strengths of this trial, implementation challenges may arise. We anticipate some difficulties in recruiting participants experiencing traumatic stress in busy clinic settings. To address this, we will work collaboratively with staff involved in HIV care at our study sites to established routine referral systems. Confidentiality and sensitivity will be paramount to the success of this trial. Participant retention is a second potential challenge. We anticipate that almost all of our participants will live in peri-urban areas and may therefore struggle to attend intervention sessions or scheduled assessments for reasons associated with informal employment, illness, and interprovincial migration. To address this, our study team will endeavor to establish collaborative relationships with participants. Moreover, we aim to enhance retention through maintenance of detailed contact information and using clinic records to align intervention-related visits with scheduled clinic appointments. Lastly, we aim to pre-empt potential vicarious trauma, compassion fatigue, or emotional distress among field staff by holding regular debriefing sessions with a clinical psychologist.

It must be acknowledged that even if this intervention is proven effective and is implemented into care settings in South Africa, it cannot address the systemic roots of sexual trauma and HIV infection. Extreme inequality linked to historic disparities impacts many upstream issues such as education, housing, and poverty. These inequalities must be addressed in order to truly improve national health outcomes [92, 93]. However, women today are living in settings that put them at increased risk for sexual violence and HIV, and they need treatment at a faster pace than is possible on a policy level. As such, we feel obligated to work towards improving current mental health needs and clinical HIV outcomes.

In summary, this trial is expected to yield important new information on psychologically informed intervention models that benefit the mental health and clinical outcomes of WLH with histories of sexual trauma. The proposed ImpACT + intervention, with its focus on building coping skills to address traumatic stress and engagement in HIV care and treatment, could have widespread impact on the health and wellbeing of individuals and communities in sub-Saharan Africa.

### Trial status

Enrollment of participants commenced in February 2021 and is expected to run through to November 2023.

## Data Availability

An anonymized (permanently de-identified) dataset will be prepared for public sharing.

## Abbreviations

AIDS: Acquired immune deficiency syndrome
ANC: Antenatal care
ART: Antiretroviral therapy
ARV: Antiretroviral
CFIR: Consolidated Framework for Implementation Research
CIDI: Composite International Diagnostic Interview
COVID-19: Coronavirus disease 2019
CUIMC: Columbia University and Irving Medical Center
DBS: Dried blood spot
DSMB: Data Safety and Monitoring Board
ENACT: Enhancing Assessment of Common Therapeutic factors
HIV: Human immunodeficiency virus
HREC: Human Research Ethics Committees
ImpACT: Improving AIDS Care after Trauma
ImpACT +: Improving AIDS Care after Trauma plus
IRB: Institutional Review Board
mhGAP: WHO Mental Health Gap Action Programme
NIMH: National Institutes for Mental Health
NHLS: National Health Laboratory Service
PCL-5: PTSD Checklist-Civilian version of the DSM-5
PMTCT: Prevention of mother-to-child transmission
PST: Problem-solving therapy
PTSD: Post-traumatic stress disorder
RA: Research Assistant
REDCap: Research Electronic Data Capture
UCT: University of Cape Town
WHO: World Health Organization
WLH: Women living with HIV

## References

[1] Seedat S (2012). Interventions to improve psychological functioning and health outcomes of HIV-infected individuals with a history of trauma or PTSD. Curr HIV/AIDS Rep.

[2] Machtinger EL, Wilson TC, Haberer JE, Weiss DS (2012). Psychological trauma and PTSD in HIV-positive women: a meta-analysis. AIDS Behav.

[3] LeGrand S, Reif S, Sullivan K, Murray K, Barlow ML, Whetten K (2015). A review of recent literature on trauma among individuals living with HIV. Curr HIV/AIDS Rep.

[4] Hatcher AM, Smout EM, Turan JM, Christofides N, Stöckl H (2015). Intimate partner violence and engagement in HIV care and treatment among women. AIDS.

[5] Gass JD, Stein DJ, Williams DR, Seedat S (2011). Gender differences in risk for intimate partner violence among south african adults. J Interpers Violence.

[6] Leserman J, Pence BW, Whetten K, Mugavero MJ, Thielman NM, Swartz MS (2007). Relation of lifetime trauma and depressive symptoms to mortality in HIV. Am J Psychiatry.

[7] Cohen MH, Cook JA, Grey D, Young M, Hanau LH, Tien P (2004). Medically eligible women who do not use HAART: the importance of abuse, drug use, and race. Am J Public Health.

[8] Anderson JC, Campbell JC, Glass NE, Decker MR, Perrin N, Farley J (2018). Impact of intimate partner violence on clinic attendance, viral suppression and CD4 cell count of women living with HIV in an urban clinic setting. AIDS Care - Psychol Socio-Medical Asp AIDS/HIV.

[9] Aaron E, Criniti S, Bonacquisti A, Geller PA (2013). Providing sensitive care for adult HIV-infected women with a history of childhood sexual abuse. J Assoc Nurses AIDS Care.

[10] Pence BW, Mugavero MJ, Carter TJ, Leserman J, Thielman NM, Raper JL (2012). Childhood trauma and health outcomes in HIV-infected patients: an exploration of causal pathways. J Acquir Immune Defic Syndr.

[11] Jina R, Thomas LS (2013). Health consequences of sexual violence against women. Best Pract Res Clin Obstet Gynaecol.

[12] Whetten K, Whetten RA, Ostermann J, Itemba D (2008). Trauma, anxiety and reported health among HIV-positive persons in Tanzania and the US Deep South. AIDS Care.

[13] Blashill AJ, Perry N, Safren SA (2011). Mental health: a focus on stress, coping, and mental illness as it relates to treatment retention, adherence, and other health outcomes. Curr HIV/AIDS Rep.

[14] Mugavero M, Ostermann J, Whetten K, Leserman J, Swartz M, Stangl D (2006). Barriers to antiretroviral adherence: the importance of depression, abuse, and other traumatic events. AIDS Patient Care STDS.

[15] Wingood GM, DiClemente RJ, Seth P (2013). Improving health outcomes for IPV-exposed women living with HIV. JAIDS J Acquir Immune Defic Syndr.

[16] Siemieniuk RAC, Krentz HB, Miller P, Woodman K, Ko K, Gill MJ (2013). The clinical implications of high rates of intimate partner violence against HIV-positive women. JAIDS J Acquir Immune Defic Syndr.

[17] Marinda E, Zungu N, Chikovore J, Mthembu J, Magampa M, Mathentamo Q (2021). Association between ART adherence and mental health: results from a national HIV sero-behavioural survey in South Africa. AIDS Behav.

[18] Jewkes R, Abrahams N (2002). The epidemiology of rape and sexual coercion in South Africa: an overview. Soc Sci Med.

[19] Jewkes R, Penn-Kekana L, Levin J, Ratsaka M, Schrieber M (2001). Prevalence of emotional, physical and sexual abuse of women in three South African provinces. S Afr Med J.

[20] Seedat M, Van Niekerk A, Jewkes R, Suffla S, Ratele K (2009). Violence and injuries in South Africa: prioritising an agenda for prevention. Lancet.

[21] Martin L, Kagee A (2011). Lifetime and HIV-related PTSD among persons recently diagnosed with HIV. AIDS Behav.

[22] Olley BO, Gxamza F, Seedat S, Theron H, Stein DJ, Taljaard J (2004). Psychopathology and coping in recently diagnosed HIV/AIDS patients - the role of gender. South African J Psychiatry.

[23] Myer L, Smit J, Le RL, Parker S, Stein DJ, Seedat S (2008). Common mental disorders among HIV-infected individuals in South Africa: prevalence, predictors, and validation of brief psychiatric rating scales. AIDS Patient Care STDS.

[24] Watt MH, Dennis AC, Choi KW, Ciya N, Joska JA, Robertson C (2017). Impact of sexual trauma on HIV care engagement: perspectives of female patients with trauma histories in Cape Town. South Africa AIDS Behav.

[25] Brief DJ, Bollinger AR, Vielhauer MJ, Berger-Greenstein JA, Morgan EE, Brady SM (2004). Understanding the interface of HIV, trauma, post-traumatic stress disorder, and substance use and its implications for health outcomes. AIDS Care - Psychol Socio-Medical Asp AIDS/HIV.

[26] Seedat S, Stein DJ, Carey PD (2005). Post-traumatic stress disorder in women: epidemiological and treatment issues. CNS Drugs.

[27] Spies G, Konkiewitz EC, Seedat S (2018). Incidence and persistence of depression among women living with and without HIV in South Africa: a longitudinal study. AIDS Behav.

[28] Cohen MA, Alfonso CA, Hoffman RG, Milau V, Carrera G (2001). The impact of PTSD on treatment adherence in persons with HIV infection [1]. Gen Hosp Psychiatry.

[29] Rooks-Peck CR, Adegbite AH, Wichser ME, Ramshaw R, Mullins MM, Higa D (2018). Mental health and retention in HIV care: a systematic review and meta-analysis. Heal Psychol.

[30] Robbertz AS, Ishiekwene MN, Hucks OL, Armistead L (2021). The impact of trauma on South African women with HIV: The role of anxiety and physical symptomology. African J AIDS Res.

[31] Dunkle KL (2004). Prevalence and patterns of gender-based violence and revictimization among women attending antenatal clinics in Soweto. South Africa Am J Epidemiol.

[32] Takuva S, Brown AE, Pillay Y, Delpech V, Puren AJ (2017). The continuum of HIV care in South Africa: Implications for achieving the second and third UNAIDS 90–90-90 targets. AIDS.

[33] Joint United Nations Programme on HIV/AIDS (UNAIDS). The gap report. Geneva: UNAIDS; 2014. p. 422.

[34] Gass JD, Stein DJ, Williams DR, Seedat S (2011). Gender differences in risk for intimate partner violence among South African adults. J Interpers Violence.

[35] Petersen Williams P, Brooke-Sumner C, Joska J, Kruger J, Vanleeuw L, Dada S (2020). Young South African women on antiretroviral therapy perceptions of a psychological counselling program to reduce heavy drinking and depression. Int J Environ Res Public Health.

[36] Verhey R, Chibanda D, Brakarsh J, Seedat S (2016). Psychological interventions for post-traumatic stress disorder in people living with HIV in Resource poor settings: a systematic review. Trop Med Int Heal.

[37] Sikkema KJ, Ranby KW, Meade CS, Hansen NB, Wilson PA, Kochman A (2013). Reductions in traumatic stress following a coping intervention were mediated by decreases in avoidant coping for people living with HIV/AIDS and childhood sexual abuse. J Consult Clin Psychol.

[38] Sikkema KJ, Hansen NB, Kochman A, Tarakeshwar N, Neufeld S, Meade CS (2007). Outcomes from a group intervention for coping with HIV/AIDS and childhood sexual abuse: reductions in traumatic stress. AIDS Behav.

[39] Myers B, Carney T, Browne FA, Wechsberg WM (2018). Development of a trauma-informed substance use and sexual risk reduction intervention for young South African women. Patient Prefer Adherence.

[40] Meffert SM, Neylan TC, McCulloch CE, Blum K, Cohen CR, Bukusi EA, et al. Interpersonal psychotherapy delivered by nonspecialists for depression and posttraumatic stress disorder among Kenyan HIV–positive women affected by gender-based violence: Randomized controlled trial. PLoS Med. 2021;18:1–22. 10.1371/journal.pmed.1003468. 10.1371/journal.pmed.1003468 PMC7799784 33428625

[41] Berghoff CR, Gratz KL, Portz KJ, Pinkston M, Naifeh JA, Evans SD (2018). The role of emotional avoidance, the patient–provider relationship, and other social support in ART adherence for HIV+ individuals. AIDS Behav.

[42] Simoni JM, Ng MT (2000). Trauma, coping, and depression among women with HIV/AIDS in New York City. AIDS Care.

[43] Ehrenkranz P, Rosen S, Boulle A, Eaton JW, Ford N, Fox MP (2021). The revolving door of HIV care: Revising the service delivery cascade to achieve the UNAIDS 95–95-95 goals. PLOS Med.

[44] Lippman SA, El Ayadi AM, Grignon JS, Puren A, Liegler T, Venter WDF, et al. Improvements in the South African HIV care cascade: findings on 90–90–90 targets from successive population-representative surveys in North West Province. J Int AIDS Soc 2019;22. 10.1002/jia2.25295. 10.1002/jia2.25295 PMC6562149 31190460

[45] Sikkema KJ, Mulawa MI, Robertson C, Watt MH, Ciya N, Stein DJ (2018). Improving AIDS Care After Trauma (ImpACT): pilot outcomes of a coping intervention among HIV-infected women with sexual trauma in South Africa. AIDS Behav.

[46] Sikkema KJ, Choi KW, Robertson C, Knettel BA, Ciya N, Knippler ET (2018). Development of a coping intervention to improve traumatic stress and HIV care engagement among South African women with sexual trauma histories. Eval Program Plann.

[47] Sikkema KJ, Wilson PA, Hansen NB, Kochman A, Neufeld S, Ghebremichael MS (2008). Effects of a coping intervention on transmission risk behavior among people living with HIV/AIDS and a history of childhood sexual abuse. JAIDS J Acquir Immune Defic Syndr.

[48] Meade CS, Drabkin AS, Hansen NB, Wilson PA, Kochman A, Sikkema KJ (2010). Reductions in alcohol and cocaine use following a group coping intervention for HIV-positive adults with childhood sexual abuse histories. Addiction.

[49] Rathod S, Persaud A, Naeem F, Pinninti N, Tribe R, Eylem O (2020). Culturally adapted interventions in mental health: global position statement. World Cult Psychiatry Res Rev.

[50] Endale T, Qureshi O, Ryan GK, Esponda GM, Verhey R, Eaton J (2020). Barriers and drivers to capacity-building in global mental health projects. Int J Ment Health Syst.

[51] Safren SA, O’cleirigh C, Andersen LS, Magidson JF, Lee JS, Bainter SA (2021). Treating depression and improving adherence in HIV care with task-shared cognitive behavioural therapy in Khayelitsha, South Africa: a randomized controlled trial. J Int AIDS Soc.

[52] Petersen I, van Rensburg A, Kigozi F, Semrau M, Hanlon C, Abdulmalik J (2019). Scaling up integrated primary mental health in six low- and middle-income countries: obstacles, synergies and implications for systems reform. BJPsych Open.

[53] Damschroder LJ, Aron DC, Keith RE, Kirsh SR, Alexander JA, Lowery JC (2009). Fostering implementation of health services research findings into practice: a consolidated framework for advancing implementation science. Implement Sci.

[54] Curran GM, Bauer M, Mittman B, Pyne JM, Stetler C (2012). Effectiveness-implementation hybrid designs: combining elements of clinical effectiveness and implementation research to enhance public health impact. Med Care.

[55] Seekings J. Economy, society and municipal services in Khayelitsha. Report for the Commission of Inquiry into Allegations of Police Inefficiency in Khayelitsha and a breakdown in relations between the community and the police in Khayelitsha, Centre for Social Science Research, University of Cape Town. 2013. p. 27.

[56] Stinson K, Goemaere E, Coetzee D, van Cutsem G, Hilderbrand K, Osler M, Hennessey C, Wilkinson L, Patten G, Cragg C, Mathee S. Cohort profile: the Khayelitsha antiretroviral programme, Cape town, South Africa. Int J Epidemiol. 2017;46(2):e21-. 10.1093/ije/dyw057. 10.1093/ije/dyw057 27208042

[57] Statistics South Africa. General Household Survey. Pretoria; 2019. 183 p. Report No.: P0318.

[58] National Department of Health. National consolidated guidelines for the prevention of mother-to-child transmission of HIV (PMTCT) and the management of HIV in children, adolescents and adults. Pretoria: Department of Health; 2015.

[59] World Health Organization (1990). Composite International Diagnostic Interview (CIDI) World Health Organization.

[60] Bernstein DP, Stein JA, Newcomb MD, Walker E, Pogge D, Ahluvalia T (2003). Development and validation of a brief screening version of the Childhood Trauma Questionnaire. Child Abus Negl.

[61] Breslau N, Peterson EL, Kessler RC, Schultz LR (1999). Short screening scale for DSM-IV posttraumatic stress disorder. Am J Psychiatry.

[62] Sheehan DV, Lecrubier Y, Sheehan KH, Janavs J, Weiller E, Keskiner A, Dunbar GC. The Mini International Neuropsychiatric Interview (MINI). A short diagnostic structured interview: reliability and validity according to the CIDI. Eur Psychiatry. 1997;12(5):224–31. 10.1016/S0924-9338(97)83296-8.

[63] Sorsdahl K, Stein DJ, Corrigall J, Cuijpers P, Smits N, Naledi T (2015). The efficacy of a blended motivational interviewing and problem solving therapy intervention to reduce substance use among patients presenting for emergency services in South Africa: a randomized controlled trial. Subst Abuse Treat Prev Policy.

[64] Van't Hof E, Stein DJ, Marks I, Tomlinson M, Cuijpers P. The effectiveness of problem solving therapy in deprived South African communities: results from a pilot study. BMC Psychiatry. 2011;11(1):1–8.10.1186/1471-244X-11-156. 10.1186/1471-244X-11-156 PMC3201896 21961801

[65] Sikkema KJ, Watt MH, Drabkin AS, Meade CS, Hansen NB, Pence BW (2010). Mental health treatment to reduce HIV transmission risk behavior: a positive prevention model. AIDS Behav.

[66] Sikkema KJ, Mulawa MI, Robertson C, Joska JA (2018). Improving AIDS Care After Trauma (ImpACT): Pilot outcomes of a coping intervention among HIV-infected women with sexual trauma in South Africa. AIDS Behav.

[67] Lazarus RS, Folkman S. Stress, appraisal, and coping. New York: Springer; 1984.

[68] Chesney MA, Chambers DB, Taylor JM, Johnson LM, Folkman S (2003). Coping effectiveness training for men living with HIV: results from a randomized clinical trial testing a group-based intervention. Psychosom Med.

[69] Bass JK, Annan J, Murray SMI, Kaysen D, Griffiths S, Cetinoglu T (2013). Controlled trial of psychotherapy for congolese survivors of sexual violence. N Engl J Med.

[70] Wickersham K, Colbert A, Caruthers D, Tamres L, Martino A, Erlen JA (2011). Assessing fidelity to an intervention in a randomized controlled trial to improve medication adherence. Nurs Res.

[71] Kolko DJ, Lindhiem O (2014). Introduction to the special series on booster sessions and long-term maintenance of treatment gains. J Abnorm Child Psychol.

[72] Kohrt BA, Ramaiya M, Rai S, Bhardwaj A, Jordans M. Development of a scoring system for non-specialist ratings fo clinical competence in global mental health: a qualitative process evaluation of the Enhancing Assessment of Common Therapeutic Factors (ENACT) scale. Glob Ment Heal. 2015;2:1–16. 10.1017/gmh.2015.21. 10.1017/gmh.2015.21 PMC5269630 28593049

[73] World Health Organisation (2016). Problem Management Plus (PM+): individual psychological help for adults impaired by distress in communities exposed to adversity.

[74] Wilson IB, Lee Y, Michaud J, Fowler FJ, Rogers WH (2016). Validation of a new three-item self-report measure for medication adherence. AIDS Behav.

[75] Chesney MA, Ickovics JR, Chambers DB, Gifford AL, Neidig J, Zwickl B, Wu AW. Self-reported adherence to antiretroviral medications among participants in HIV clinical trials: the AACTG adherence instruments. Patient Care Committee & Adherence Working Group of the Outcomes Committee of the Adult AIDS Clinical Trials Group (AACTG). AIDS Care. 2000;12(3):255–66. 10.1080/09540120050042891. 10.1080/09540120050042891 10928201

[76] Weathers FW, Litz BT, Keane TM, Palmieri PA, Marx BP, Schnurr PP. The PTSD Checklist for DSM-5 (PCL-5). National Center for PTSD. Available at www.ptsd.va.gov.

[77] Namir S, Wolcott DL, Fawzy FI, Alumbaugh MJ (1987). Coping with AIDS: psychological and health implications. J Appl Soc Psychol.

[78] Folkman S, Lazarus RS (1988). The relationship between coping and emotion: Implications for theory and research. Soc Sci Med.

[79] Carver CS (1997). You want to measure coping but your protocol’ too long: consider the brief cope. Int J Behav Med.

[80] Kotze M, Visser M, Makin J, Sikkema K, Forsyth B (2013). The coping strategies used over a two-year period by HIV-positive women who had been diagnosed during pregnancy. AIDS Care - Psychol Socio-Medical Asp AIDS/HIV.

[81] Hansen NB, Harrison B, Fambro S, Bodnar S, Heckman TG, Sikkema KJ (2013). The structure of coping among older adults living with HIV/AIDS and depressive symptoms. J Health Psychol.

[82] Harris PA, Taylor R, Thielke R, Payne J, Gonzalez N, Conde JG (2009). Research electronic data capture (REDCap)-A metadata-driven methodology and workflow process for providing translational research informatics support. J Biomed Inform.

[83] Zou GY, Donner A (2013). Extension of the modified Poisson regression model to prospective studies with correlated binary data. Stat Methods Med Res.

[84] Preacher KJ (2015). Advances in mediation analysis: a survey and synthesis of new developments. Annu Rev Psychol.

[85] Guest G, MacQueen K, Namey E (2014). Applied Thematic. Analysis.

[86] Miles MB, Michael Huberman A, Saldaña J. Qualitative data analysis. A methods sourcebook. Los Angeles: SAGE Publications; 2014.

[87] Carpenter JR, Kenward MG. Missing data in randomised controlled trials: a practical guide. Health Technology Assessment Methodology Programme, Birmingham. 2007. p. 199. https://researchonline.lshtm.ac.uk/id/eprint/4018500.

[88] UNAIDS. Global AIDS update 2019—communities at the centre. Geneva: UNIADS; 2019.

[89] Nakimuli-Mpungu E, Musisi S, Smith CM, Von Isenburg M, Akimana B, Shakarishvili A (2021). Mental health interventions for persons living with HIV in low- and middle-income countries: a systematic review. J Int AIDS Soc.

[90] Waldron EM, Burnett-Zeigler I, Wee V, Ng YW, Koenig LJ, Pederson AB, et al. Mental health in women living with HIV: the unique and unmet needs. J Int Assoc Providers AIDS Care. 2021;20:1–18. 10.1177/2325958220985665. 10.1177/2325958220985665 PMC7829520 33472517

[91] Keynejad RC, Dua T, Barbui C, Thornicroft G (2018). WHO Mental Health Gap Action Programme (mhGAP) Intervention Guide: a systematic review of evidence from low and middleincome countries. Evid Based Ment Health.

[92] Belli P, Matsebula T, Ndhlalambi M, Ngarachu M. A brief profile of the status of health and the health system in South Africa. Washington, D.C.: World Bank, Washington, DC; 2018. 10.1596/30036.

[93] Lund C, Brooke-Sumner C, Baingana F, Baron EC, Breuer E, Chandra P (2018). Social determinants of mental disorders and the Sustainable Development Goals: a systematic review of reviews. The Lancet Psychiatry.

